# Coherent Surface Plasmon Hole Burning via Spontaneously Generated Coherence

**DOI:** 10.3390/molecules26216497

**Published:** 2021-10-27

**Authors:** Habibur Rahman, Hazrat Ali, Rafi Ud Din, Iftikhar Ahmad, Mahidur R. Sarker, Sawal Hamid Md Ali

**Affiliations:** 1 Department of Physics, University of Malakand Chakdara Dir Lower, Malakand 23050, Pakistan; habibur@yahoo.com (H.R.); ahma5532@gmail.com (I.A.); 2 Department of Physics, Abbottabad University of Science and Technology, Havelian 22500, Pakistan; 3 School of Physics, Huazhong University of Science and Technology, 1037 Luoyu Road, Wuhan 430074, China; rafisolo15@gmail.com; 4 Department of Physics, VC Gomal University, Dera Ismail Khan 29340, Pakistan; 5 Institute of IR 4.0, Unverisity Kebangsaan Malaysia, Bangi 43600, Malaysia; 6 Department of Electrical, Electronic and Systems Engineering, Faculty of Engineering and Built Environment, University Kebangsaan Malaysia, Bangi 43600, Malaysia

**Keywords:** plasmon hole burning, spontaneously generated coherence, silver medium

## Abstract

Surface plasmon (SP)—induced spectral hole burning (SHB) at the silver-dielectric interface is investigated theoretically. We notice a typical lamb dip at a selective frequency, which abruptly reduces the absorption spectrum of the surface plasmons polaritons (SPP). Introducing the spontaneous generated coherence (SGC) in the atomic medium, the slope of dispersion becomes normal. Additionally, slow SPP propagation is also noticed at the interface. The spectral hole burning dip is enhanced with the SGC effect and can be modified and controlled with the frequency and intensity of the driving fields. The SPP propagation length at the hole-burning region is greatly enhanced under the effect of SGC. A propagation length of the order of 600 µm is achieved for the modes, which is a remarkable result. The enhancement of plasmon hole burning under SGC will find significant applications in sensing technology, optical communication, optical tweezers and nano-photonics.

## 1. Introduction

The coupled state of electromagnetic radiation and surface free charges in metals results in a wave, propagating at the interface known as surface plasmon polaritons (SPPs). SPPs have been widely used to confine the EM-field in sub-wavelength region [1,2,3,4]. Owing to their sub-wavelength nature, SPPs can provide a basic platform for nanotechnology. SPP have useful applications in spectroscopy, optical circuits, photo detectors, polarizers, nanolithography, lasers and amplifiers, bio and chemical sensors, nanophotonics and many others [5,6,7,8,9,10,11,12,13,14]. SPP excited at the interface lies in the visible or in infrared frequency range. SPP propagates along the interface, where its intensity decays exponentially on both sides of the interface. The exploration of SPP at the quantum level plays a key role in the designing of plasmonic devices [15,16]. Plasmonic nanostructures have a sub-wavelength nature, which can be used to trap light in a thin absorber layer. Metal nano-particles, having different shapes, sizes, and surface densities, can be used as absorption enhancers in perovskite solar cells. The idea of absorption enhancement may be useful in the development of plasmonic-based photovoltaic devices. The control and modified SPP propagation on the nanometer scale play a vital role in nanophotonics devices, near-field optics, data storage devices, and solar cells [17,18,19,20]. Owing to the strong field confinement capability and local field enhancement ability, the SPPs at the interfaces provide a platform for the optical sensors. Typically, a surface plasmon resonance (SPR) structure is more suitable for the detection of larger biomolecules, due to its longer decay length. However, localized surface Plasmon resonance (LSPR) has a shorter decay length and is suitable for detecting smaller biomolecules [21]. The SPR through Ti films can be used for infrared thermal imaging, and information detection [22]. The characteristics of a light pulse passing through a metal–dielectric–metal (MDM) waveguide coupled with a ring-type cavity were investigated [23]. The plasmon-induced transmission through the MDM waveguide coupled to a slot cavity was used to demonstrate the mode selection and filtering tunability of SPPs [24,25].

When a probe light pulse excites a material with inhomogeneous absorption, a spectral hole forms in the absorption spectrum, called spectral hole burning (SHB). The phenomenon of SHB has been studied in various nonlinear atomic systems [26,27,28]. Coherent hole burning was achieved in the absorption spectrum, using Kerr nonlinearity in the atomic system [29]. Doppler broadening and Kerr nonlinearity were used to generate SHB in different atomic systems. Doppler broadening is an incoherent effect, which depends on the temperature of the medium [30]. In recent years, spontaneously generated coherence (SGC) has attracted much attention of researchers. The phenomenon of coherent population trapping (CPT) [31], the enhancement and degrading of group speed of light and electromagnetically induced transparency (EIT) were investigated under the effect of SGC in the atomic media [32,33,34]. Similarly, lasing without inversion (LWI), a quantum coherent effect, was also realized in the atomic system [35]. Quantum interference can be used significantly in the field of both linear as well as nonlinear optics [36]. The dipole moments of the quantum states of the atomic media causes SGC. SGC can be efficiently used in the manipulation of physical phenomena, such as control over the fields generating micro waves in an anisotropic media [37]. Several works on the experimental realization of SGC in different media were reported [38]. The optical trap technique can be used for the realization of SGC in $Rb^{87}$. The relative expansion in the spectrum due to the Doppler effect is known as Doppler broadening. Agarwal et al. achieved a slow group speed of up to $c/10^{3}$ with an incoherent effect of Doppler broadening [39]. Slow light at the spectral hole burning region with Doppler effect were also investigated [40].

Keeping in view the significance of SPP hole burning in modern optical devices, this paper is focused on investigating SHB at the metal/dielectric interface. Further, we study SHB in the dispersion spectrum of SPP with various spectroscopic parameters. We investigate the enhancement in the SPP hole burning, due to the SGC at the silver–rubidium interface. The SPP propagation length in the hole burnt regions is studied, which is further enhanced under the SGC. The achieved propagation length is 600 μm at the interface, which is six times larger than the results obtained under the Kerr effect [41]. Our enhanced results will find significant application in sensing technology, optical tweezers and photovoltaic devices.

## 2. Materials and Methods

**Figure 1:**
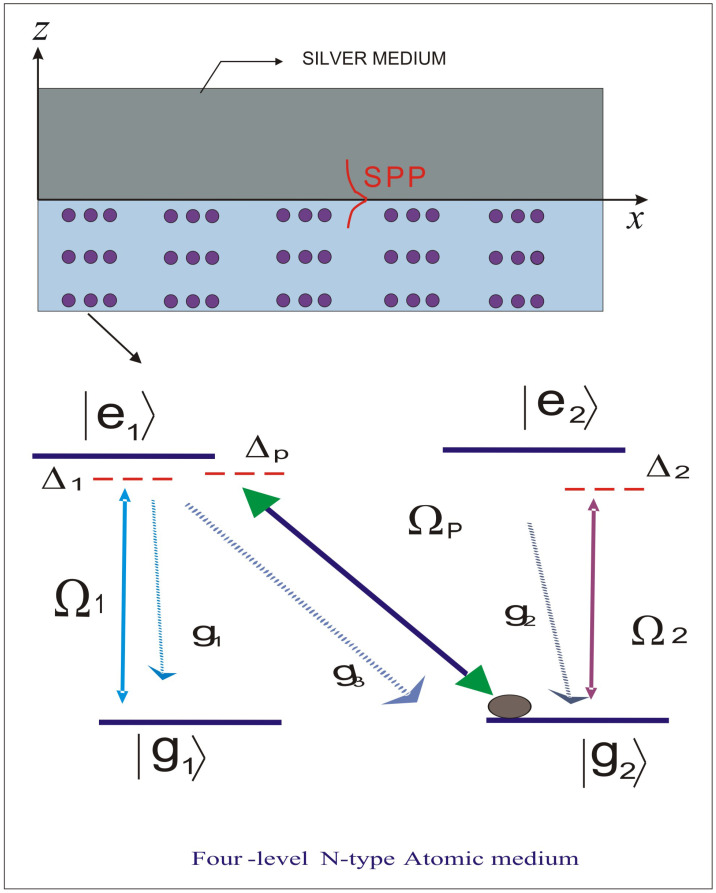
Schematic of interface between a metal and four-level N-type atomic system. The upper half space (Z > 0) includes metal (silver), whereas the lower half space (Z < 0) contains the four-level atomic medium. The probe field is applied between state $|g_{2}\rangle$ and $|e_{1}\rangle$, which further excites the SPP on the interface.

We consider the interface between a metal and four-level N-type atomic system to demonstrate the interesting phenomenon of SHB for SPP. In Figure 1, the upper half space includes metal (silver), whereas the lower half space contains the four-level atomic medium. SPP are shown at the interface between a metal and dielectric media. The four-level N-type atomic medium contains two degenerate lower and excited states. A strong field $E_{1}$ with Rabi frequency $\Omega_{1}$ is applied to the couple, $|g_{1}\rangle$ and $|e_{1}\rangle$. Similarly, $|g_{2}\rangle$ and $|e_{2}\rangle$ are coupled by another coherent beam with Rabi frequency $\Omega_{2}$. A perturbing field of Rabi frequency $\Omega_{p}$ causes the initial perturbation in the states $|g_{2}\rangle$ and $|e_{1}\rangle$, as can be seen in Figure 1. The four-level N-type atomic system can be realized in $Rb^{87}$. This system was considered for different purposes in Refs. [30,42,43]. The Hamiltonian in the interaction form for the above system can be expressed as follows:

(1) $$\begin{array}{ccc} H_{I} & = & -\frac{\hbar }{2}\Omega_{1}e^{-i(\Delta_{1})t}|g_{1}\rangle \langle e_{1}|\\ & & -\frac{\hbar }{2}\Omega_{2}e^{-i(\Delta_{2})t}|g_{2}\rangle \langle e_{2}|\\ & & -\frac{\hbar }{2}\Omega_{P}e^{-i(\Delta_{P})t}|g_{2}\rangle \langle e_{1}|+H.c. \end{array}$$

While $\Delta_{1}=\omega_{g_{1}e_{1}}-\omega_{1}$, $\Delta_{p}=\omega_{g_{2}e_{1}}-\omega_{P}$ and $\Delta_{2}=\omega_{g_{2}e_{2}}-\omega_{2}$. The dynamics of the system can be described by the master equation as follows:

(2) $$\frac{d}{dt}\rho_{t}=-\frac{i}{\hbar }\left[H_{I},\rho_{t}\right]-\frac{1}{2}\gamma_{ij}\sum \left(\alpha^{†}\alpha \rho_{t}+\rho_{t}\alpha^{†}\alpha -2\alpha \rho_{t}\alpha^{†}\right),$$

where $\alpha^{†}$ and α are ladder operators. From Equation (2), we can derive the coupling rate equations as follows:

(3) $$\begin{array}{ccc} {\dot{\rho }}_{e_{1}g_{2}}^{\left(1\right)} & = & \left[i\Delta_{p}-\gamma_{1}-\gamma_{2}\right]\rho_{e_{1}g_{2}}^{\left(1\right)}+i\Omega_{1}\rho_{g_{1}g_{2}}^{\left(1\right)}\\ & & -i\Omega_{P}\left(\rho_{g_{2}g_{2}}^{\left(0\right)}-\rho_{e_{1}e_{1}}^{\left(0\right)}\right)-i\Omega_{2}\rho_{e_{1}e_{2}}^{\left(1\right)}\\ & & -2P\sqrt{\gamma_{2}\gamma_{3}}\rho_{e_{22}}^{\left(1\right)}, \end{array}$$

(4) $$\begin{array}{ccc} {\dot{\rho }}_{g_{1}g_{2}}^{\left(1\right)} & = & \left[-i\left(\Delta_{1}-\Delta_{P}\right)\right]\rho_{g_{1}g_{2}}^{\left(1\right)}+i\Omega_{1}\rho_{e_{1}g_{2}}^{\left(1\right)}\\ & & -i\Omega_{P}\rho_{e_{1}g_{1}}^{\left(1\right)}-i\Omega_{2}\rho_{g_{1}e_{2}}^{\left(1\right)}, \end{array}$$

(5) $$\begin{array}{ccc} {\dot{\rho }}_{e_{1}e_{2}}^{\left(1\right)} & = & [-i\left(\Delta_{P}-\Delta_{2}\right)\rho_{e_{1}e_{2}}^{\left(1\right)}+\\ & & i\Omega_{1}\rho_{g_{1}e_{2}}^{\left(1\right)}+i\Omega_{P}\rho_{g_{2}e_{2}}^{\left(1\right)}-i\Omega_{2}\rho_{e_{1}g_{2}}^{\left(1\right)}, \end{array}$$

(6) $$\begin{array}{ccc} {\dot{\rho }}_{g_{1}e_{2}}^{\left(1\right)} & = & [-i\left(\Delta_{1}-\Delta_{P}+\Delta_{2}\right)-\\ & & \gamma_{2}]\rho_{g_{1}e_{2}}^{\left(1\right)}+i\Omega_{1}\rho_{e_{1}e_{2}}^{\left(1\right)}-i\Omega_{2}\rho_{g_{1}e_{1}}^{\left(1\right)}, \end{array}$$

(7) $$\begin{array}{ccc} {\dot{\rho }}_{e_{1}e_{1}}^{\left(1\right)} & = & \left[-\gamma_{1}-\gamma_{2}\right]\rho_{e_{1}e_{1}}^{\left(1\right)}+i\Omega_{1}\rho_{g_{1}e_{1}}^{\left(1\right)}-i\Omega_{1}\rho_{e_{1}g_{1}}^{\left(1\right)}, \end{array}$$

(8) $$\begin{array}{ccc} {\dot{\rho }}_{g_{1}e_{1}}^{\left(1\right)} & = & \left[-i\Delta_{1}-\gamma_{1}\right]\rho_{g_{1}e_{1}}^{\left(1\right)}+i\Omega_{1}\rho_{e_{1}e_{1}}^{\left(1\right)}, \end{array}$$

and

(9) $$\begin{array}{ccc} {\dot{\rho }}_{e_{1}g_{1}}^{\left(1\right)} & = & \left[i\Delta_{1}-\gamma_{1}\right]\rho_{e_{1}g_{1}}^{\left(1\right)}-i\Omega_{1}\rho_{e_{1}e_{1}}^{\left(1\right)}. \end{array}$$

The atoms are assumed to be prepared in the ground state $\rho_{g_{2}g_{2}}^{\left(0\right)}=1$. Therefore, the initial population of atoms for other states is zero, i.e., $\rho_{g_{1}g_{1}}^{\left(0\right)}=\rho_{e_{1}e_{1}}^{\left(0\right)}=\rho_{e_{2}e_{2}}^{\left(0\right)}=0$.

To derive ${\tilde{\rho }}_{g_{2}e_{1}}^{\left(1\right)}$, the following equation can be used:

(10) $$W=\int_{-\infty }^{t}e^{-X}Ydt=-X^{-1}Y.$$

Here, *W* and *Y* are column matrices, while *X* is the square matrix. The complex susceptibility of the dielectric medium can be calculated as follows:

(11) $$\begin{array}{c} \chi_{e}=\beta \frac{2i\left(\Delta_{1}+\Delta_{P}\right)\Omega_{1}^{2}+C_{11}C_{22}+\frac{\Omega_{2}^{2}}{4}}{C_{33}}, \end{array}$$

where

(12) $$\begin{array}{c} \beta =-\frac{2N\mu_{23}^{2}}{\hbar \epsilon_{0}}, \end{array}$$

(13) $$\begin{array}{c} C_{11}=i\Delta_{P}-\left(\gamma_{1}+\gamma_{2}\right), \end{array}$$

(14) $$\begin{array}{ccc} C_{22} & = & i(\Delta_{P}-\Delta_{1})\\ & & -(\gamma_{1}+\gamma_{2}+\gamma_{3}), \end{array}$$

and

(15) $$\begin{array}{ccc} C_{33} & = & \Omega_{2}^{2}4\left(\gamma_{1}+\gamma_{2}-i\Delta_{P}\right)\\ & & (\gamma_{1}+\gamma_{2}-i\Delta_{P}+\Omega_{2}^{2}-\Omega_{1}^{2}\\ & & +16(\gamma_{3}+i\Delta_{1}-i\Delta_{P}). \end{array}$$

In the above equations, *N* denotes the atomic density of the sample, and $\mu_{23}$ represents the electric dipole moment. *P* is the SGC factor, which depends on the non-orthogonality of the dipole moments. The SGC effect can be achieved in the control field as $\Omega_{1}=\Omega_{0}\sqrt{1-P^{2}}$, $\Omega_{0}=4.5\gamma$ throughout the calculation.

The relative motion of atoms in a medium, due to the increase in the temperature of the medium experiences a change in the frequency of the incident beam of light. The change in the frequency of the incident light can be efficiently used in the modification of the optical properties of the atomic medium. The relative shift of the frequency can be significantly used in manipulation of the absorption and dispersion spectra of SPP at the interfaces. The Doppler broadened electric susceptibility under Maxwell distribution can be written as follows:

(16) $$\begin{array}{c} \chi_{e}\left(d\right)=\frac{1}{\sqrt{2\pi D^{2}}}\int_{-\infty }^{\infty }\chi_{e}e^{-\frac{{\left(\kappa \nu \right)}^{2}}{2D^{2}}}d\left(\kappa \nu \right). \end{array}$$

The detunings of the levels are affected as $\Delta_{P}+kv\eta_{P}$, $\Delta_{1}+kv\eta_{1}$ and $\Delta_{2}+kv\eta_{2}$, where *k* and *v* are the wave number and relative velocity of atomic motion in the presence of Doppler effect. Similarly, $\eta_{P},\eta_{1}$ and $\eta_{2}$ are the relative directions of the propagating fields in the atomic system. If $\eta_{i=1,2}=1=\eta_{P}$, it indicates parallel propagations of the control and probe fields. On the other hand, if $\eta_{i=1,2}=-1=\eta_{P}$, then it shows counter propagation. In the above equation, $D=\sqrt{2K_{B}T_{k}/m}$ is the Doppler width, where $K_{B}$ is the Boltzman constant, *m* is the atomic mass and $T_{k}$ is the absolute temperature.

The probe field generates the SPP spectrum at the metal/dielectric interface for which the dispersion relation can be written as follows:

(17) $$\begin{array}{c} k_{sp}=\frac{\omega }{c}\sqrt{\frac{\varepsilon_{e}^{d}\varepsilon_{m}}{\left(\varepsilon_{e}^{d}+\varepsilon_{m}\right)}}. \end{array}$$

Here, ($\varepsilon_{e}^{d}$) is the dielectric of the atomic system and can be written as follows:

(18) $$\begin{array}{c} \varepsilon_{e}^{d}=1+\chi_{e}^{d}, \end{array}$$

while the dielectric constant of silver is obtained from Drude’s model as follows:

(19) $$\begin{array}{c} \varepsilon_{m}=\varepsilon_{\infty }-\frac{\omega_{sp}^{2}}{\omega^{2}+i\Gamma \omega }. \end{array}$$

Here, $\omega_{sp}$ represents the plasma frequency, $\varepsilon_{\infty }$ describes the contribution of bound electrons, while Γ is the scattering rate. The group refractive index for the SPP can be written as $n_{g}=n_{r}+\omega \frac{\partial n_{r}}{\partial \omega }$, which can be used to derive the group velocity of SPP at the interface. Further, $k_{sp}=k_{0}n_{r}$, where $k_{0}=\frac{\omega }{c}$ is the wave number of the probe field. The propagation length of SPP is related with imaginary part of $k_{spp}$ and is written as $L_{sp}=1/2Im\left(k_{sp}\right)$.

## 3. Results

We investigate the interesting features of surface plasmon–induced hole burning at the silver–dielectric interface, where the dielectric medium consists of a large number of four-level atoms. The SGC effect is introduced in the atomic system, which can further enhance the spectral profile of plasmon-induced hole burning. The decay γ is scaled as a standard for the other spectroscopic parameters and is taken to be 1 MHz. To simplify our results, we put $\hbar ,\mu_{0},\epsilon_{0}=1$. The atomic density of the sample is taken to be $N=2\times 10^{12}$ atoms/cm${}^{3}$. The relative velocity of the atoms and temperature of the medium are kept constant.

In our model, the spectral hole dip can be achieved in the absorption, dispersion, propagation length and in the group index of the plasmons. The dispersion of SPP $k_{sp}$ is a complex response function, where the imaginary part is related to the absorption. The real and imaginary part of the $k_{sp}$ is related to the dispersion and absorption spectra of SPP.

**Figure 2:**
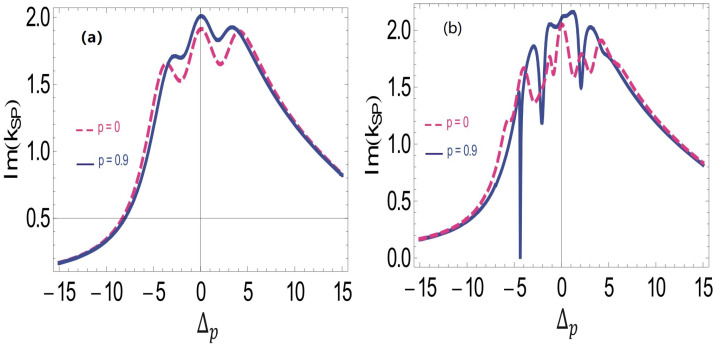
SPP absorption spectrum at the interface with SGC effect vs $\Delta_{P}$, such that (**a**) γ = 1 MHz, $\gamma_{1}=\gamma_{2}=1\gamma$, $\gamma_{3}=0\gamma$, $\Delta_{1}=0.01\gamma$, $\Delta_{2}=0\gamma$, $\eta_{1}=0$, $\eta_{2}=0$, $\omega_{ps}=1.5\times 10^{16}$, $\omega =3.6\times 10^{15}$, $\Omega_{2}=2.5\gamma$ and $\Omega_{0}=4.5\gamma$, $\hbar ,\mu_{0},\epsilon_{0}=1$, and $N=2\times 10^{12}$ atoms/cm${}^{3}$; (**b**) γ = 1 MHz, $\gamma_{1}=\gamma_{2}=1\gamma$, $\gamma_{3}=0\gamma$, $\Delta_{1}=0.01\gamma$, $\Delta_{2}=0\gamma$, $\eta_{1}=1$, $\eta_{2}=1$, D=0.9, $\omega_{ps}=1.5\times 10^{16}$, $\omega =3.6\times 10^{15}$, $\Omega_{2}=2.5\gamma$, $\Omega_{0}=4.5\gamma$, $\hbar ,\mu_{0},\epsilon_{0}=1$, and $N=2\times 10^{12}$ atoms/cm${}^{3}$.

To investigate the absorption spectrum of SPP at the interface in the presence of co-propagating probe and control fields, we trace the plot between the imaginary part of $k_{sp}$ and probe field detuning $\Delta_{p}$. The absorption peak is observed at the resonance point with no burnt regions by considering the SGC effect in the system. We notice an enhanced absorbtion spectrum of the SPP by considering the SGC effect in the system; see the solid curve of Figure 2a. Hence, we see no plasmon hole burnt region at the interface between the silver and the Doppler-broadened atomic medium when both the control and probe field propagate in the same direction with the thermal atoms. In Figure 2b, the plot is traced for the imaginary part of $k_{sp}$ and control field detuning $\Delta_{p}$ at the interface of a N-type atomic medium and silver metal when both the fields counter propagate, i.e., $\eta_{1},\eta_{2}=-1,1$. When there is no SGC effect in the atomic levels, the absorption spectrum is maximal at the resonance point and we observe small, multiple hole burns. The increase in the detuning of the probe field reduces the absorption of SPP at the interface. The SGC factor introduced in the system produces multiple absorption dips of SPP at the interface. The effect of SGC strongly influences the absorption spectrum of SPP and produces the three spectral hole burning regions. We report slow SPP in the hole burning region, which can further be used for manipulation of SPP at the interface. The dispersion slope becomes normal for the hole-burning region, which corresponds to the positive group index of the medium.

**Figure 3:**
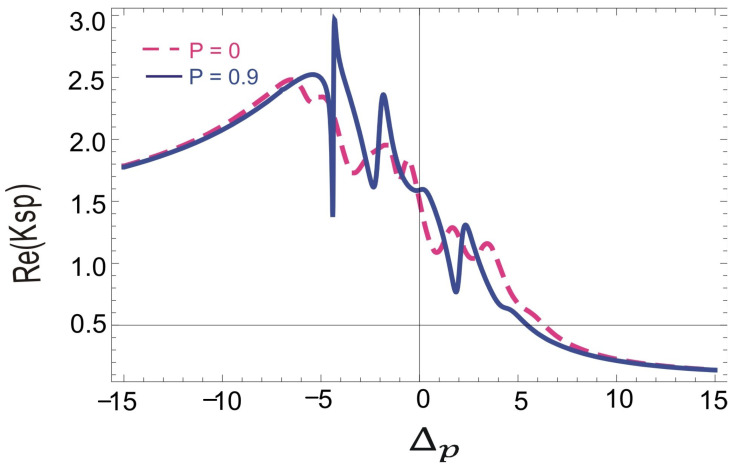
Dispersion spectrum of slow light medium with Doppler broadening and SGC effect vs. $\Delta_{P}$ such that γ = 1 MHz, $\gamma_{1}=\gamma_{2}=1\gamma$, $\gamma_{3}=0\gamma$, $\Delta_{1}=0.01\gamma$, $\Delta_{2}=0\gamma$, $\eta_{1}=1$, $\eta_{2}=-1$, D=0.9, $\omega_{ps}=1.5\times 10^{16}$, $\omega =3.6\times 10^{15}$, $\Omega_{2}=2.5\gamma$, $\Omega_{0}=4.5\gamma$, $\hbar ,\mu_{0},\epsilon_{0}=1$, and $N=2\times 10^{12}$ atoms/cm${}^{3}$.

Next, we investigate the plasmons-induced hole burnt in the dispersion spectrum of SPP at the interface of a four-level N-type Doppler-broadening atomic medium and silver metal as presented in Figure 3. Dispersion spectrum of SPP varies both with the SGC factor and with the control field detuning. When the SGC factor is zero, the dispersion spectrum is small near the resonance point. Increasing the control field detuning in the positive domain results in the decrease in the dispersion of SPP at the metal dielectric interface. However, in the negative domain of the control field, detuning the decrease in the dispersion of SPP is negligibly small as shown in the dashed pink line of Figure 3. Further, to manipulate the plasmon hole burning in the dispersion spectrum, we introduced the effect of SGC. A sharp lamb dip can be seen in the dispersion of SPP at the $\Delta_{2}=-5\gamma$. SPP are slowed down in the hole burnt regions at the interface of our proposed system. The dispersion of SPP saturates at the plasmon hole burning. Moreover, the dispersion of SPP also varies with the control field detuning as shown with the solid blue spectral line of Figure 3. The slope of the dispersion spectrum is normal, revealing slow SPP propagation near the interface.

**Figure 4:**
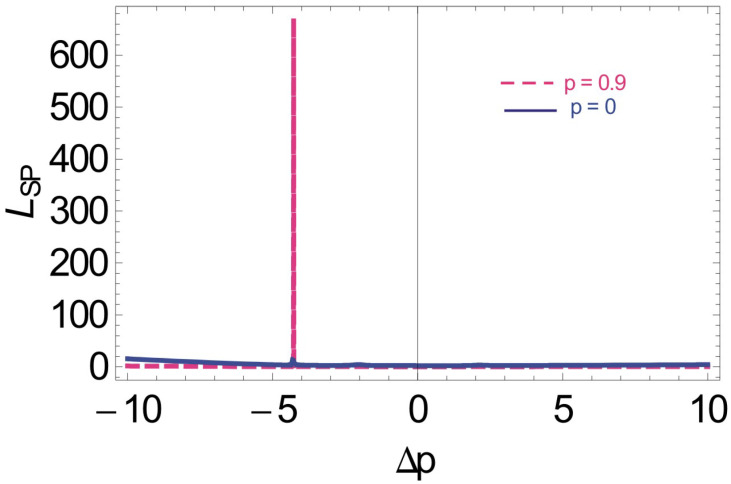
Propagation length of SPP vs. $\Delta_{P}$ with Doppler broadening and SGC effect, such that γ = 1 MHz, $\gamma_{1}=\gamma_{2}=\gamma_{3}=0.1\gamma$, D=0.9, $\Delta_{1}=\Delta_{2}=0\gamma$, $\eta_{1}=1$, $\eta_{2}=-1$, $\omega_{ps}=1.5\times 10^{16}$, $\omega =3.6\times 10^{15}$, $\Omega_{2}=2.5\gamma$, $\Omega_{0}=4.5\gamma$, $\hbar ,\mu_{0},\epsilon_{0}=1$, and $N=2\times 10^{12}$ atoms/cm${}^{3}$.

To present the SPP confinement on the interface of an N-type atomic medium and a metal, we measure the propagation length of SPP on both sides of the interface. Interestingly, we observe a plasmon hole burning, where the propagation length of SPP is measured. When the SGC factor is 0, the propagation length of SPP is almost 50 μm; however, the propagation length quickly enhances to 600 μm for the SGC factor of 0.9, as shown in the dashed pink line of Figure 4. Since SPP decays exponentially on both sides of the interface with a very short decay time, probably in the femto second, it is very important to degrade the speed of SPP at the interface. Plasmon hole burning can be efficiently modified to manipulate slow SPP, typically at the interface of silver and atomic media. The decay time of SPP for silver is relatively larger than gold or aluminum, etc. The results obtained for the plasmon hole burning under the SGC are five to six times enhanced, compared to the results obtained under the Kerr effect [41].

**Figure 5:**
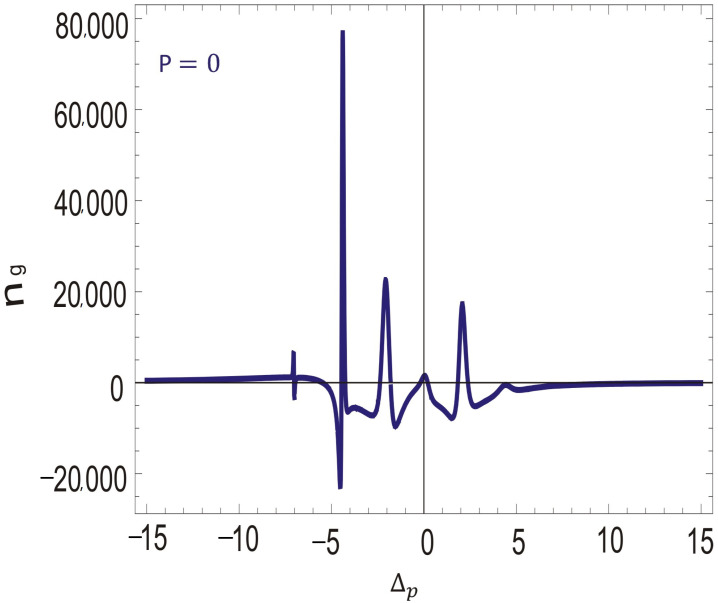
Group index of SPP versus $\Delta_{p}$ with Doppler broadening and SGC Effect, such that γ = 1 MHz, $\gamma_{1}=\gamma_{2}=\gamma_{3}=0.1\gamma$, D=0.9, $\Delta_{1}=\Delta_{2}=0\gamma$, $\eta_{1}=1$, $\eta_{2}=-1$, $\omega_{ps}=1.5\times 10^{16}$, $\omega =3.6\times 10^{15}$, $\Omega_{2}=2.5\gamma$, $\Omega_{0}=4.5\gamma$, p = 0, $\hbar ,\mu_{0},\epsilon_{0}=1$, and $N=2\times 10^{12}$ atoms/cm${}^{3}$.

In Figure 5, the plot is traced for the group refractive index of SPP with the probe field detuning. The dispersion relation of SPP is related with the group refractive index. The group index of SPP largely enhances at the interface with SGC. Near the resonance point, the group index is small. However, the group refractive index enhances quickly in the negative domain of probe field detuning. A spectral hole appears at $\Delta_{P}=-5\gamma$. The group index is positive and large at the spectral hole burnt region, revealing an enhancement in the slow group velocity of SPP. SPP propagates on both sides of the interface with a very short decay time. Slow propagation of SPP will efficiently modify the plasmonic-based devices. Further, the group refractive index of SPPs is very sensitive to the SGC in our proposed system. A slight increase in the SG coherence causes a large enhancement in the group index as shown in Figure 5.

**Figure 6:**
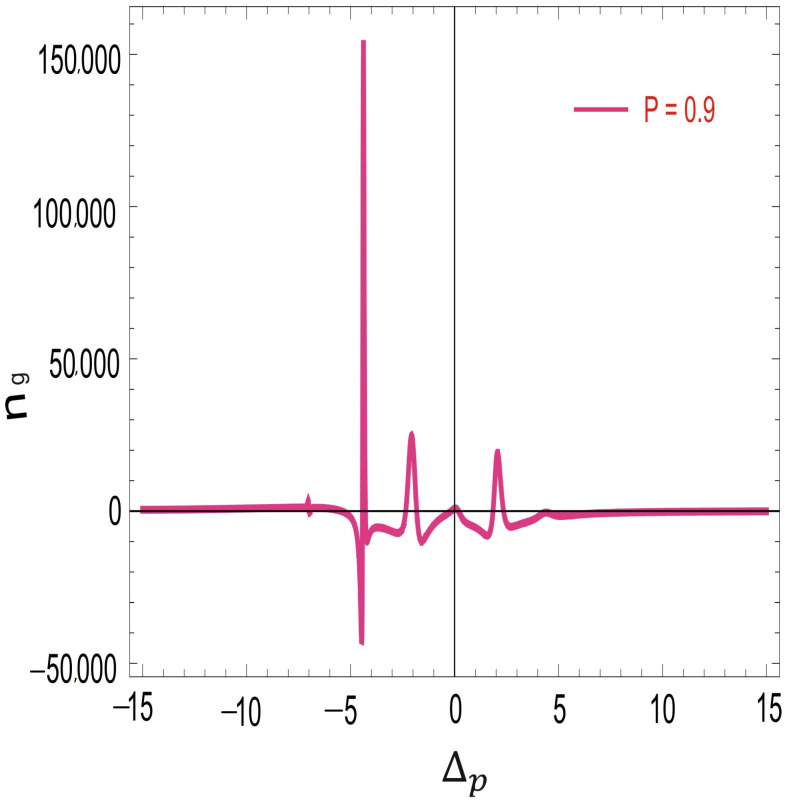
Group index of SPP vs. $\Delta_{p}$ with Doppler broadening and SGC effect such that γ = 1 MHz, $\gamma_{1}=\gamma_{2}=\gamma_{3}=0.1\gamma$, D=0.9, $\Delta_{1}=\Delta_{2}=0\gamma$, $\eta_{1}=1$, $\eta_{2}=-1$, $\omega_{ps}=1.5\times 10^{16}$, $\omega =3.6\times 10^{15}$, $\Omega_{2}=2.5\gamma$, $\Omega_{0}=4.5\gamma$, p = 0.9, $\hbar ,\mu_{0},\epsilon_{0}=1$, and $N=2\times 10^{12}$ atoms/cm${}^{3}$.

Next, to study the strong dependence of group refractive index on the SGC factor at the interface, we explain Figure 6.

When the SGC is slightly increased, the group index largely enhances in the positive domain. The group index enhances from 80,000 to 150,000 under SGC at the interface of silver and atomic media. These results provide a new degree of freedom to manipulate slow SPP in a hole burnt region via SGC. The decay time of SPP for the silver interface is larger than that of other noble metals, which further enhances due to the enhancement in the slow group velocity. The control over SPP via SGC in a hole burnt region may be very helpful in optical tweezers, sensors technology, solar panels, optical circuits and SPP-based optical switches.

## 4. Conclusions

In summary, plasmon hole burning at the silver–dielectric (four-level N-type atomic medium) interface was investigated by varying the spontaneously generated coherence (SGC) effect. The absorption, dispersion and propagation length of the SPP hole burning region were modified and controlled under the SGC effect. Due to the fast decay rate of an SPP, it needed to be slowed down considerably. The control over surface plasmon hole burning was efficiently used to slow down the SPP at the interface. The group index and quantum confinement of the SPP were greatly enhanced at the hole burning region with the SGC effect. Most remarkably, a propagation length of about 600 μm for the SPP was noticed, which can be of particular interest in plasmonics. The enhanced results for plasmon hole burning may be helpful in plasmon-based sensing technology, optical communication, wave-guides, spectroscopy, radiation guiding and optical tweezers.

## Data Availability

The data presented in this study are available on reasonable request from the corresponding author.
